# Supramolecular tholos-like architecture constituted by archaeal proteins without functional annotation

**DOI:** 10.1038/s41598-020-58371-2

**Published:** 2020-01-30

**Authors:** Maho Yagi-Utsumi, Arunima Sikdar, Chihong Song, Jimin Park, Rintaro Inoue, Hiroki Watanabe, Raymond N. Burton-Smith, Toshiya Kozai, Tatsuya Suzuki, Atsuji Kodama, Kentaro Ishii, Hirokazu Yagi, Tadashi Satoh, Susumu Uchiyama, Takayuki Uchihashi, Keehyoung Joo, Jooyoung Lee, Masaaki Sugiyama, Kazuyoshi Murata, Koichi Kato

**Affiliations:** 1 Exploratory Research Center on Life and Living Systems (ExCELLS), National Institutes of Natural Sciences, Okazaki, Aichi 444-8787 Japan; 2 Institute for Molecular Science (IMS), National Institutes of Natural Sciences, Okazaki, Aichi 444-8787 Japan; 3 SOKENDAI (The Graduate University for Advanced Studies), Okazaki, Aichi 444-8787 Japan; 4 Graduate School of Pharmaceutical Sciences, Nagoya City University, Nagoya, Aichi 467-8603 Japan; 5 National Institute for Physiological Sciences, National Institutes of Natural Sciences, Okazaki, Aichi 444-8787 Japan; 6 School of Computational Sciences, Korea Institute for Advanced Study, Seoul, 02455 Republic of Korea; 7 Institute for Integrated Radiation and Nuclear Science, Kyoto University, Kumatori, Osaka 590-0494 Japan; 8 Department of Physics, Nagoya University, Nagoya, Aichi 464-8602 Japan; 9 Department of Biotechnology, Graduate School of Engineering, Osaka University, Suita, Osaka 565-0871 Japan; 10 Center for Advanced Computation, Korea Institute for Advanced Study, Seoul, 02455 Republic of Korea; 11 Beijing Computational Science Research Center, Haidian District, Beijing, 10084 China

**Keywords:** Cryoelectron microscopy, Supramolecular assembly

## Abstract

Euryarchaeal genomes encode proteasome-assembling chaperone homologs, PbaA and PbaB, although archaeal proteasome formation is a chaperone-independent process. Homotetrameric PbaB functions as a proteasome activator, while PbaA forms a homopentamer that does not interact with the proteasome. Notably, PbaA forms a complex with PF0014, an archaeal protein without functional annotation. In this study, based on our previous research on PbaA crystal structure, we performed an integrative analysis of the supramolecular structure of the PbaA/PF0014 complex using native mass spectrometry, solution scattering, high-speed atomic force microscopy, and electron microscopy. The results indicated that this highly thermostable complex constitutes ten PbaA and ten PF0014 molecules, which are assembled into a dumbbell-shaped structure. Two PbaA homopentameric rings correspond to the dumbbell plates, with their N-termini located outside of the plates and C-terminal segments left mobile. Furthermore, mutant PbaA lacking the mobile C-terminal segment retained the ability to form a complex with PF0014, allowing 3D modeling of the complex. The complex shows a five-column tholos-like architecture, in which each column comprises homodimeric PF0014, harboring a central cavity, which can potentially accommodate biomacromolecules including proteins. Our findings provide insight into the functional roles of Pba family proteins, offering a novel framework for designing functional protein cages.

## Introduction

Proteins forming supramolecular assemblies perform complex functions in cells—best exemplified by the proteasome. The central part of this large enzyme complex shows a cylindrical architecture, termed the 20S proteasome, which acts as proteolytic machinery for selective protein degradation, thereby regulating various biological processes^1–3^. In eukaryotes, the formation of this architecture is not a spontaneous process but is assisted by several other proteins termed proteasome-assembling chaperones, including the homologous Pba1 and Pba2 proteins, which form a heterodimer and thereby serve as molecular matchmaker and check-point for the correct arrangements of structurally homologous proteasomal subunits^4–10^. In contrast, the archaeal 20S proteasome comprises much less divergent subunits, which can self-organize into the cylindrical architecture without assembling chaperones^11,12^. Paradoxically, euryarchaeal genomes encode the Pba1/Pba2 homologs termed PbaA and PbaB. All these proteins possess a C-terminal proteasome-binding motif.

In our previous studies, we determined the crystal structures of PbaA and PbaB from *Pyrococcus furiosus*, which form a homopentamer and homotetramer, respectively, but not any hetrooligomers^13,14^. Moreover, we demonstrated that the PbaB homotetramer binds the mature 20S proteasome through its protruding C-terminal segments, thereby functioning as an ATP-independent proteasome activator^14^. In contrast, the other homolog PbaA is even more enigmatic because it cannot interact with the 20S proteasome despite the presence of the potential C-terminal proteasome-binding motif. Indeed, our previous study showed that the PbaA homopentamer almost exclusively adopts a closed conformation, with its C-terminal segments packed against the hydrophobic surface of pentameric core domains^15^.

Notably, small-angle X-ray scattering (SAXS)-based high-throughput structural analysis of proteins from *P. furiosus* revealed that PbaA interacts with PF0014, an archaeal protein without functional annotation, forming a massive complex with a radius of gyration of 55.0 Å^16^. This protein is conserved in *Euryarchaeota* species and shares no sequence similarity with the proteasomal subunits, suggesting its functional role independent of the proteasome^17^. However, the lack of sufficient structural details of the formation of the PbaA/PF0014 complex impedes the understanding the biological functions of PbaA and PF0014. Therefore, in this study, we attempted the structural characterization of the PbaA/PF0014 complex using an integrative biophysical approach implementing high-speed atomic force field microscopy (HS-AFM), native mass spectrometry (MS), electron microscopy (EM), and solution X-ray and neutron scattering.

## Results and Discussion

### Overall structure of the PbaA/PF0014 complex

**Figure 1:**
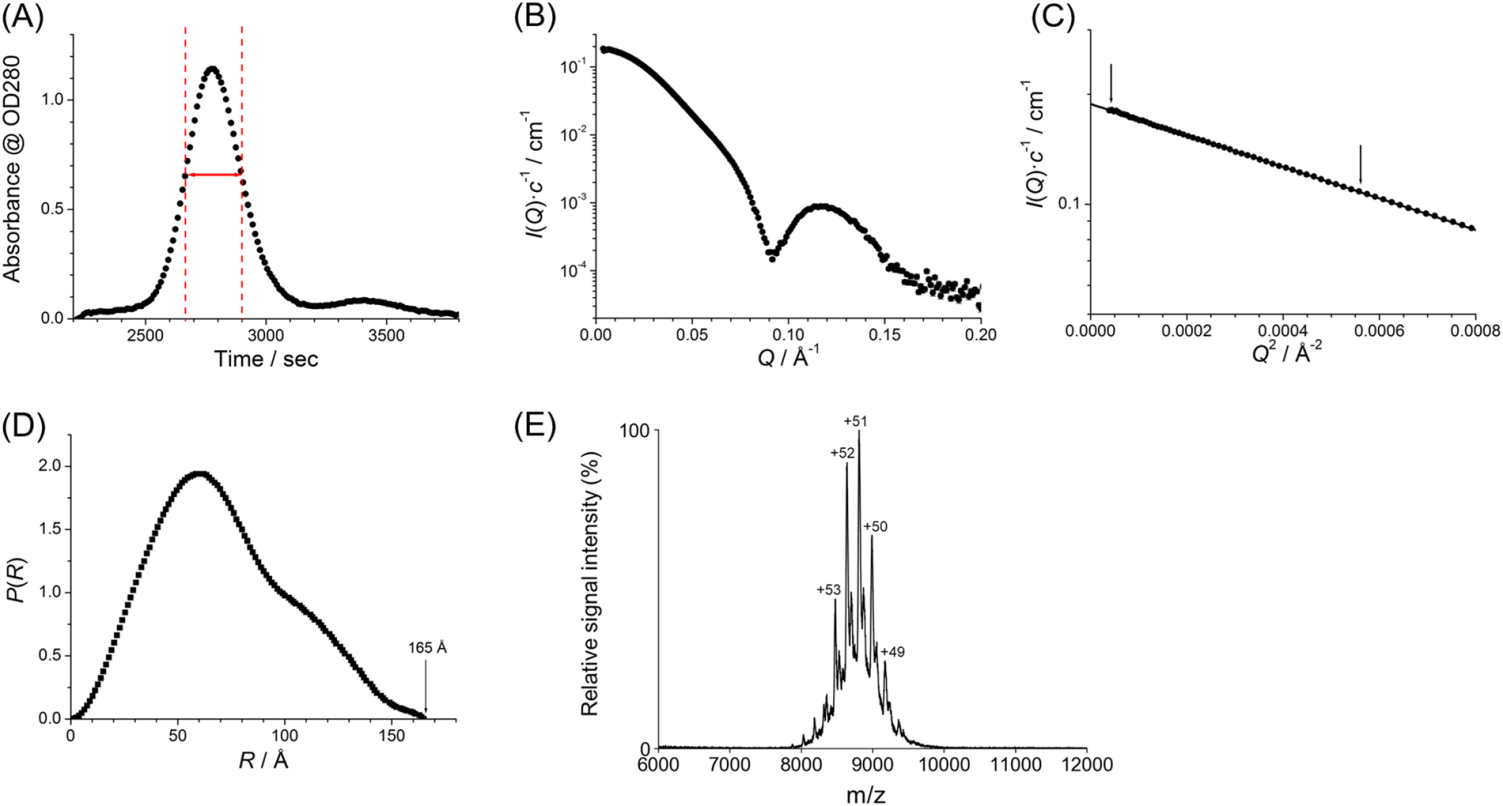
SEC-SAXS and native MS analyses. (**A**) The SEC chart of the PbaA/PF0014 complex. We selected a region of full width at half maximum of the main peak from *t* = 2664 to *t* = 2900 s, indicated by red lines, for averaging the SAXS profile. (**B**) Averaged scattering profile. (**C**) Guinier plot indicating the range for the least square fitting by arrows and (**D**) distance distribution function, *P*(*r*), of the averaged SAXS profile. (**E**) Mass spectra of the mixtures of PbaA and PF0014 at a 1:1 molar ratio under non-denaturing conditions.

We prepared PbaA and PF0014 as bacterially expressed recombinant proteins with an N-terminal hexahistidine tag. Equimolar mixtures of these proteins were subjected to size-exclusion chromatography (SEC)-SAXS, which revealed a high-molecular weight species with an estimated radius of gyration (*R*_g_) of 54.6 Å and maximum dimension (*D*_max_) of 165 Å (Fig. 1A–D and Supplementary Figure S1). This result is consistent with the findings of a previously reported SAXS-based high-throughput structural analysis^16^. To determine the stoichiometry of the PbaA/PF0014 complex, we performed native MS analysis (Fig. 1E, Supplementary Figure S2). The molecular mass of the complex was 449,192.2 ± 41.5 Da, corresponding to a 10:10 PbaA/PF0014 complex, with a calculated mass of 449,151.2 Da. Using SEC, we confirmed that PbaB did not form a complex with PF0014 (Supplementary Figure S1). As expected, the differential scanning calorimetry (DSC) data indicated that PbaA and its complex with PF0014 were thermostable with denaturing temperature above 100 °C (Supplementary Figure S3).

**Figure 2:**
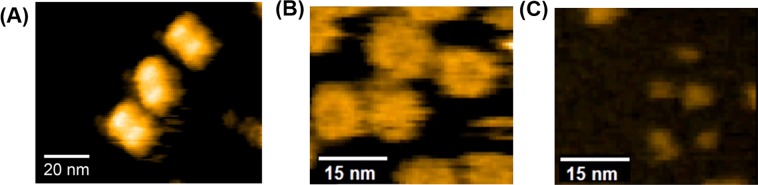
HS-AFM analyses. Typical HS-AFM images of (**A**) the PbaA/PF0014 complex, (**B**) the PbaA pentamer, and (**C**) PF0014.

**Figure 3:**
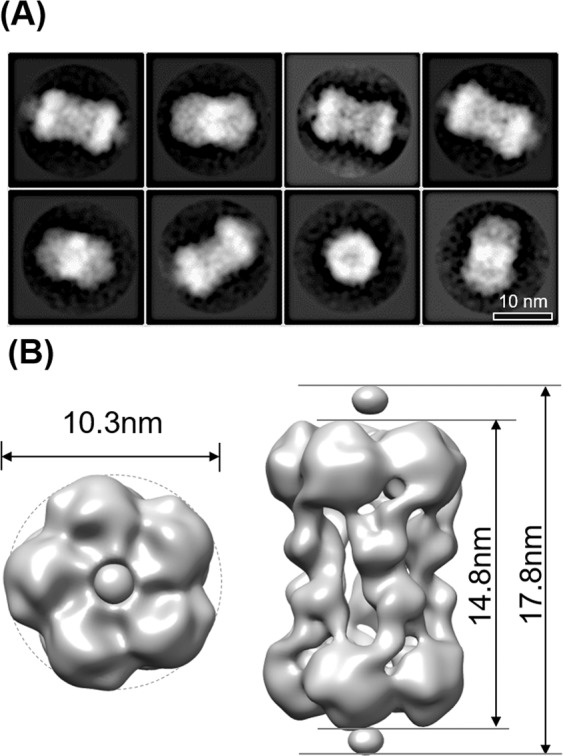
Negative stain EM analysis of the PbaA/PF0014 complex. (**A**) Two-dimensional averaged images by negative stain EM. (**B**) Three-dimensional reconstructions of the top (left) and side (right) views by single particle analysis.

We performed HS-AFM experiments to obtain information on the overall structure of the PbaA/PF0014 complex in solution. HS-AFM images showed that the PbaA/PF0014 complex assumed a dumbbell-shaped structure (Fig. 2A, Supplementary Movie S1), while the isolated forms of PbaA and PF0014 exhibited a pentameric ring and monomeric structure, respectively (Fig. 2B,C, Supplementary Movies S2 and S3). We performed negative stain EM and single-particle analysis to provide 3D structural details of the PbaA/PF0014 complex, which confirmed the dumbbell-shaped structure of the complex (Fig. 3, Supplementary Table S1).

### Spatial arrangements of PbaA and PF0014 in the complex

**Figure 4:**
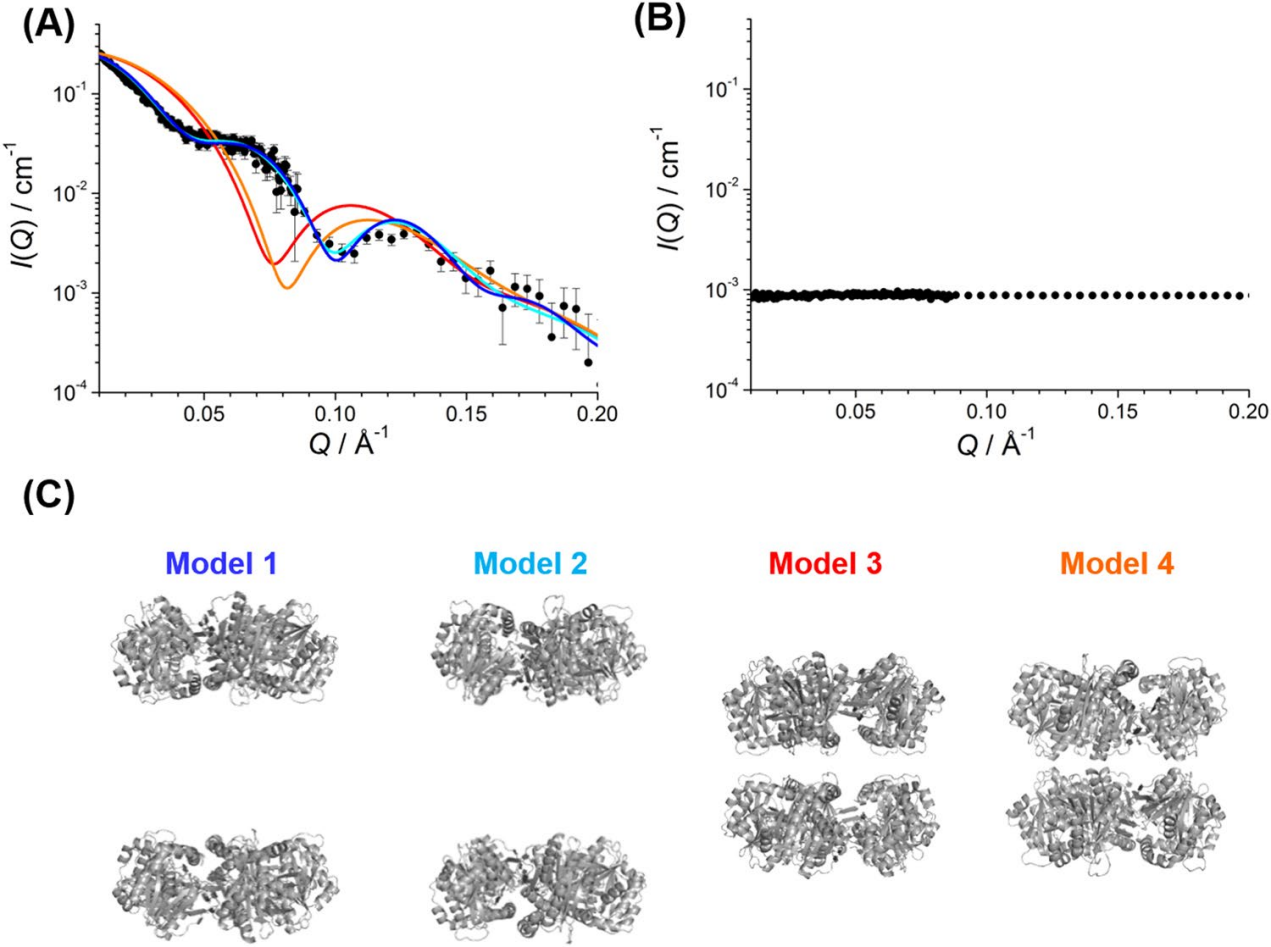
iCM-SANS analyses. (**A**) SANS profiles of the 75%-deuterated PF0014/non-deuterated PbaA complex. Closed circles indicate the experimental data, and blue, cyan, red, and orange lines represent the theoretical profiles calculated from models 1–4, respectively. (**B**) SANS profile of the 75%-deuterated PF0014/75%-deuterated PbaA complex in 97% D_2_O. (**C**) The hypothetical position and orientation of pentameric rings of PbaA.

Next, we determined the positions of PbaA and PF0014 in the complex, assuming that the pentameric ring structure of PbaA was preserved. We performed inverse contrast-matching (iCM)-SANS^18–20^ experiments using a complex formed between 75%-deuterated PF0014 and non-deuterated PbaA for the selective observation of PbaA in D_2_O solution. We examined whether the experimentally obtained scattering profile of this complex could be reproduced using a pair of pentameric models of PbaA, which were positioned at the center or ends of the dumbbell-shaped structure in either head-to-head or tail-to-tail orientation. The experimental profile was consistent with the models in which two PbaA pentameric rings were situated at both ends of the dumbbell, although the orientation was ambiguous (Fig. 4).

**Figure 5:**
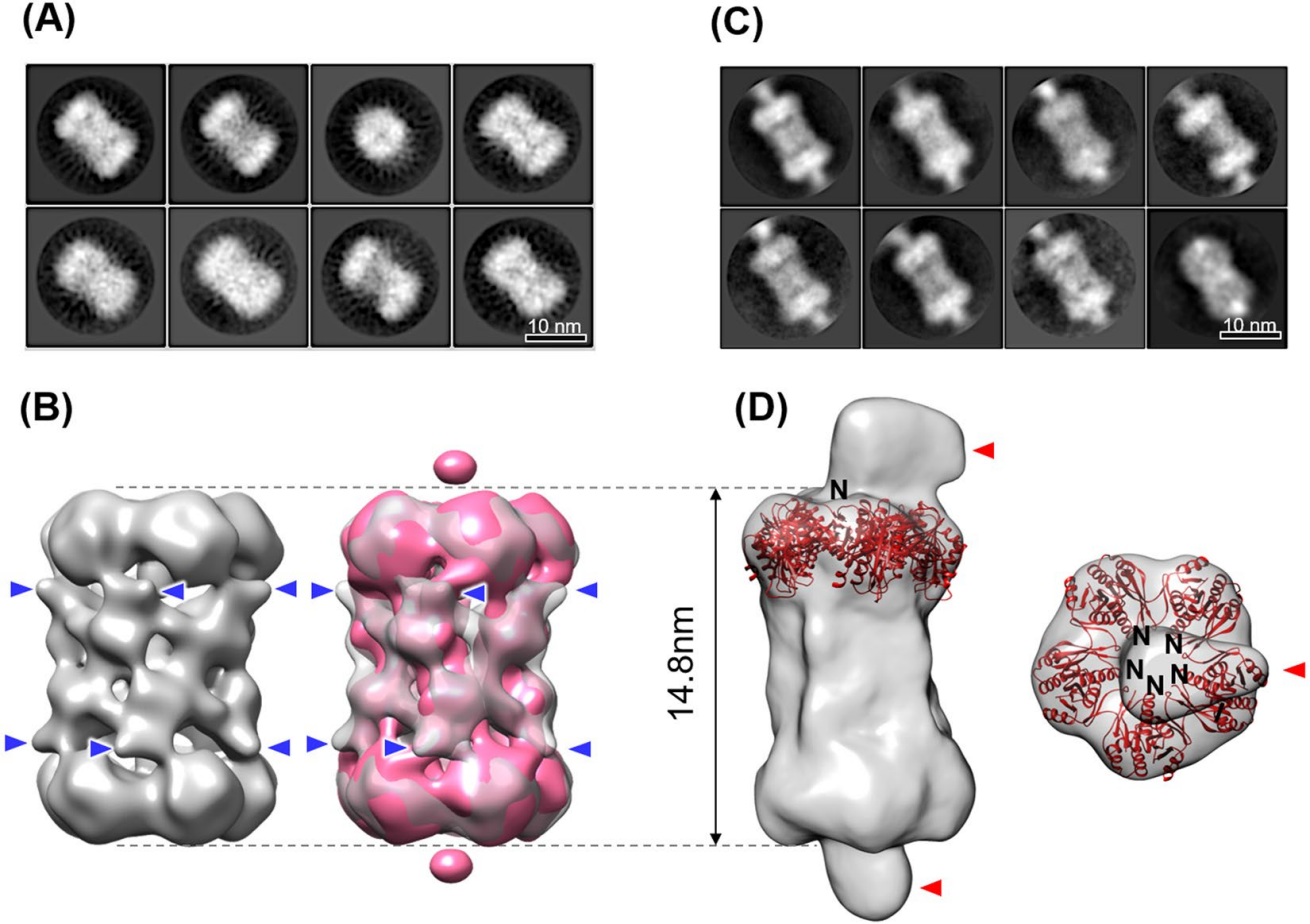
EM analyses of the PbaA/Trx-PF0014 complex and the hexahistidine-tagged PbaA/Trx-PF0014 complex with a monoclonal antibody. (**A**) Two-dimensional averaged images by negative stain EM and (**B**) three-dimensional reconstructions of the side view of the PbaA/Trx-PF0014 complex by single particle analysis. The EM map of the PbaA/PF0014 complex (Fig. 3B, magenta) was superimposed onto that of PbaA/Trx-PF0014 complex (right). Blue arrowheads indicate the spike-like extra densities. (**C**) Two-dimensional averaged images by cryo-EM and (**D**) three-dimensional reconstructions of the side view (left) and top view (right) of the hexahistidine-tagged PbaA/Trx-PF0014 complex with a monoclonal antibody directed against the histidine tag by single particle analysis. Red arrowheads indicate the extra densities originating from the anti-histidine tag antibody. The pentameric core part of the crystal structure of PbaA (PDB code: 3WZ2) was superimposed onto the EM map with indication of N-termini.

Furthermore, to probe the positions and orientations of PF0014 and PbaA, the two proteins were N-terminally tagged with thioredoxin (Trx) and hexahistidine, respectively. The EM structure of the complex formed between PbaA and Trx-tagged PF0014 (Trx-PF0014) was virtually identical to that of the native complex except for the spike-like extra densities surrounding the dumbbell-shaft center (arrowheads, Fig. 5A,B, Supplementary Table S1). These additional densities could be ascribed to the fused Trx moieties, although their volume did not perfectly match the crystal structure of Trx and was partially ambiguous presumably due to their flexibility. The HS-AFM data of the PbaA/Trx-PF0014 complex showed that the smaller lobe dynamically moved around the dumbbell-shaft region (Supplementary Movie S4). In contrast, a fluctuating lobe was not observed in the HS-AFM image of the complex between PbaA and PF0014 without the Trx moiety (Supplementary Movie S1). These results indicated that the observed smaller lobes of the PbaA/Trx-PF0014 complex correspond to the highly flexible Trx moieties. These data indicated that the ten PF0014 molecules constitute the dumbbell-shaft, with their N-termini positioned outside, nearly in a transverse plane, including the center. Using the N-terminally hexahistidine-tagged PbaA forming a complex with the Trx-tagged PF0014 and further labeled with the monoclonal antibody against the histidine tag, we confirmed the locations of PbaA pentamers as the dumbbell plates; moreover, we revealed that the N-termini of PbaA are located on the outside of the plates (arrows, Fig. 5C,D, Supplementary Table S1). Only a single antibody molecule bound each end of the complex probably due to steric hindrance because the PbaA N-termini clustered at the center of the pentamer.

### Modeling the atomic structure of the PbaA_CΔ30_/PF0014 complex

The aforementioned results suggested that the C-terminal segments of PbaA were located on the opposite side of the dumbbell plate. However, we were not able to find the densities derived from the PbaA C-terminal segments into the negative stain EM map, which is probably due to their flexible properties in the complex. In the previous study, we reported that the vast majority of the PbaA pentamer existed in a closed conformation with the packed C-terminal segments; however, it exhibited an open conformation as a minor form^15^. Hence, we examined whether these segments are involved in the formation of the PbaA/PF0014 complex. We developed a mutant PbaA by the deletion of 30 C-terminal amino acid residues (PbaA_CΔ30_) and investigated its interaction with PF0014. SEC and negative stain EM revealed that the mutant PbaA exclusively formed a double-homoheptameric structure in the absence of PF0014 (Supplementary Figure S4); intriguingly, however, it formed a complex with PF0014, similar to the full-length PbaA (Supplementary Figure S1).

**Figure 6:**
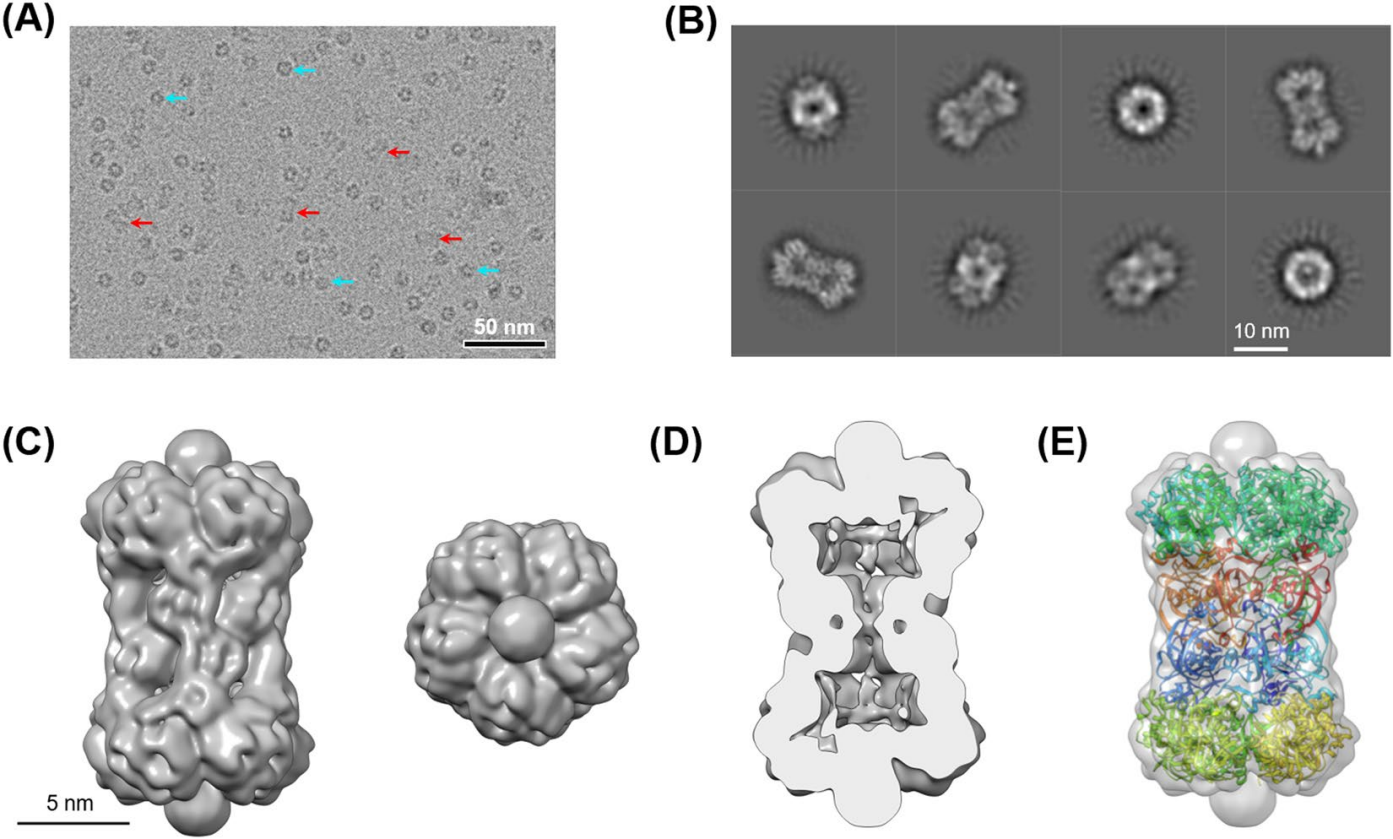
Cryo-EM analyses of the PbaA_CΔ30_/PF0014 complex. (**A**) Raw micrograph from JEM-2100F equipped with K2 Summit detector. Top views are high contrast rings (four examples are marked by cyan arrows) while side views are lower contrast (four examples are marked by red arrows). (**B**) Representative two-dimensional class averages. (**C**) Surface views of three-dimensional reconstruction of the side view (left) and top view (right) contoured at 3σ. (**D**) Long axis cross-section of the cryo-EM map allowing visualisation of internal density. (**E**) The simulated model structure of the PbaA_CΔ30_/PF0014 complex superimposed onto the transparent view of the cryo-EM map.

We structurally characterized this complex using HS-AFM and cryo-EM and confirmed the overall structural similarity between the PbaA/PF0014 and PbaA_CΔ30_/PF0014 complexes, indicating that the C-terminal segments of PbaA are not involved in its interaction with PF0014 (Fig. 6A–C, Supplementary Table S1, Movie S5). Notably, the cryo-EM densities derived from PF0014 were more clearly observed in the PbaA_CΔ30_/PF0014 complex than in the PbaA/PF0014 complex because of the elimination of the ambiguous densities caused by the mobile C-terminal segments. Consequently, we found that the PbaA/PF0014 complex shows a five-column tholos-like architecture, rather than a dumbbell-shaped one, hence harboring a large cavity inside the complex (Fig. 6D).

**Figure 7:**
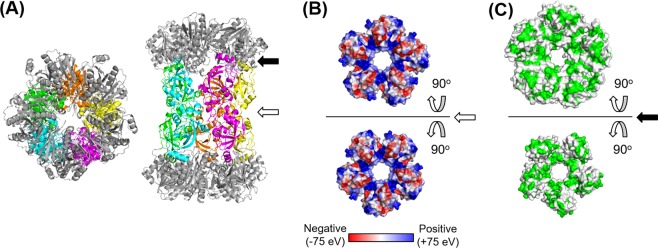
Atomic model of the PbaA_CΔ30_/PF0014 complex. (**A**) Ribbon models of the PbaA_CΔ30_/PF0014 complex. The right (top view) and left (side view) structures are related by a rotation of 90° around the horizontal axis. Two pentamers of PbaA_CΔ30_ were shown in gray. Five dimers of PF0014 were shown in yellow, orange, green, cyan, and magenta. Black and white arrows indicate the interfaces between PbaA_CΔ30_ and PF0014 and between two PF0014 protomers, respectively. (**B**) Interaction surfaces between the two PF0014 pentagons (upper and lower), shown with the electrostatic potential. Electrostatic potential was calculated and visualized using the PyMOL software. (**C**) Interaction surfaces between the PbaA_CΔ30_ pentamer (upper) and PF0014 pentagon (lower) surfaces in the model of the PbaA_CΔ30_/PF0014 complex, shown with the hydrophobic residues in green. In (**B,C**), the interaction surfaces are shown by opening the model at the white and black arrows in (**A**) respectively.

Each column may comprise two PF0014 molecules. Since PF0014 is aggregation-prone at high concentrations in the absence of PbaA, we were not able to determine the experimental structure of PF0014. Therefore, we attempted to model the tertiary structure of PF0014 to predict its interaction with PbaA based on the cryo-EM map of the PbaA_CΔ30_/PF0014 complex with an assumption that each column of the tholos corresponds to the symmetric PF0014 dimer (Figs. 6E and 7A). The most common strategy to build the atomic model in the medium resolution cryo-EM structure is to assemble and refine the structures of the individual component molecules by fitting them into the cryo-EM map in which the starting structures are set to either an experimentally known structure or a homology model. The starting structure of PbaA was set to its crystal structure (PDB ID: 3WZ2) and that of PF0014 was set to the model obtained by *in silico* protein modeling due to the lack of a known experimental structure. The final PbaA_CΔ30_/PF0014 complex model satisfied the stereochemical criteria, with a MolProbity score^21^ of 2.3 and a goodness-of-fit to the cryo-EM map with the cross-correlation coefficient of 0.93 calculated by UCSF Chimera^22^ (Fig. 6E). Our PF0014 model implied that PF0014 is a globular protein with a three-stranded anti-parallel β-sheet surrounded by five α-helices that can form a homodimer mainly via electrostatic complementarity, with the N-terminal segments placed proximal to one another (Fig. 7B and Supplementary Figure S6A). The dimeric structure of PF0014 corresponded to each column mediating the two PbaA homopentameric rings lacking the C-terminal tails, primarily through hydrophobic interactions (Fig. 7C and Supplementary Figure S6B). The model indicated mosaic distributions of the charged and hydrophobic residues on the inner surface of a three-chambered cavity (Supplementary Figure S7).

## Concluding Remarks

Our integrated data delineated the five-column tholos-like architecture of a 10:10 complex formed between two functionally unannotated proteins—PbaA and PF0014. In this architecture, ten PbaA molecules form two homopentameric rings connected by the five columns, each constituting a PF0014 dimer. In the absence of PF0014, the PbaA pentamer exhibits a closed conformation, in which the C-terminal helical segments conceal the hydrophobic surface of the pentameric core, preventing the rings from self-dimerization^13,15^. In our model, the rod-shaped PF0014 dimers interacted with the hydrophobic surface through their hydrophobic patches at both ends, in competition with the C-terminal segments of PbaA, which were consequently extruded from the pentameric ring surface and thereby become mobile in the complex. Although PF0014 alone is aggregation-prone, its multiple joint integrations with the pentavalent PbaA rings enable the formation of a thermally stable architecture.

In euryarchaeal genomes such as those of *Pyrococcus*, *Archaeoglobus*, *Methanococcus*, and *Methanocaldococcus* species, the genes encoding PbaA and PF0014 are located in the same transcription unit, presumably to ensure the formation of the 10:10 complex in cells. The tholos-like architecture of the PbaA/PF0014 complex suggests its proteasome-independent function, although the presence of the potential proteasome-binding motif at the PbaA C-terminus remains enigmatic. Despite its high thermostability, the complex harbors a large central cavity, which can potentially accommodate biomacromolecules including proteins.

Our findings highlight the versatility of the Pba family proteins in terms of their homo-oligomeric forms as well as hetero-oligomeric complex formation with different binding partners. The unique tholos-like architectures of the PbaA/PF0014 complex discovered in this study provide insight into the functional roles of these proteins. Furthermore, our research offers a novel framework for designing functional protein cages.

## Methods

### Expression and purification of PbaA

*P. furiosus* genomic DNA was provided by RIKEN BioResource Center (Japan). Expression and purification of *P. furiosus* PbaA (PF0015) was performed as described previously^13,14^. PbaA_CΔ30_ was constructed using standard genetic engineering techniques. Expression and purification of PbaA_CΔ30_ were performed according to the protocol for the purification of full-length PbaA^13^.

### Expression and purification of PF0014

*P. furiosus* PF0014 expressed with an N-terminal hexahistidine tag was purified using a Ni^2+^-immobilized affinity column (Chelating Sepharose, GE Healthcare) from *E. coli* soluble lysate and then directly subjected to complex formation with PbaA. To prepare deuterated proteins, bacterial cells were grown in an M9 minimal medium containing glucose as a mixture with 25% of isotopically natural form and 75% of fully deuterated from (1,2,3,4,5,6,6-D_7_, 98%, Cambridge Isotope Laboratories, Inc.), along with 25% H_2_O and 75% D_2_O, as previously described^23^. To determine PF0014 positions in the complex, Trx-PF0014 was used. Trx-PF0014 with an N-terminal hexahistidine tag was purified using a Ni^2+^-immobilized affinity column (Chelating Sepharose, GE Healthcare). After cleavage of the hexahistidine tag by thrombin protease, Trx-PF0014 was purified using a HiTrapQ HP anion exchange column (GE Healthcare) and HiLoad Superdex 200 column (GE Healthcare) in 20 mM Tris–HCl (pH 8.0) containing 200 mM NaCl and 2 mM dithiothreitol (DTT).

### Purification of the PbaA/PF0014 complex

For the purification of the PbaA/PF0014 complex, both recombinant proteins, PbaA (400 μM monomer) and PF0014 (400 μM monomer), were mixed at a 1:1 molar ratio and then dialyzed for 12 h against 50 mM Tris–HCl buffer (pH 8.0) containing 200 mM NaCl and 2 mM DTT. The high-molecular-weight complex was fractionated using a HiTrapQ HP anion exchange column (GE Healthcare) and HiLoad Superdex 200 column (GE Healthcare) with the same buffer conditions. The PbaA_CΔ30_/PF0014 and PbaA/Trx-PF0014 complexes were purified using the same protocol. As reference, PbaB (400 μM monomer) and PbaB (400 μM monomer) mixed with PF0014 (400 μM monomer) were subjected to SEC using the same protocol.

### Native MS

Native MS was performed as described previously^24^. The purified PbaA/PF0014 complex (20 μM) was buffer-exchanged into 150 mM ammonium acetate, pH 9.0, by passing the proteins through a Bio-Spin 6 column (Bio-Rad). The buffer-exchanged PbaA/PF0014 complex (20 μM) was 2-fold diluted by 150 mM ammonium acetate, pH 9.0, and immediately analyzed by nanoflow electrospray ionization MS using gold-coated glass capillaries made in-house (approximately 2–5 µL sample loaded per analysis). Spectra were recorded on a SYNAPT G2-S*i* HDMS mass spectrometer (Waters, Manchester, UK) in positive ionization mode at 1.33 kV with a 150-V sampling cone voltage and source offset voltage, 0-V trap and transfer collision energy, and 5-mL/min trap gas flow. The spectra were calibrated using 1 mg/mL cesium iodide and analyzed using the Mass Lynx software (Waters Milford, Massachusetts, USA).

### HS-AFM

HS-AFM images of protein complexes were acquired in the tapping mode using a laboratory-built HS-AFM apparatus^25^ and a short cantilever (Olympus: BL-AC7; 6–7-μm long, 2-μm wide, and 90-nm thick) at room temperature. The PbaA/PF0014 complex was dissolved at a concentration of 1.0–1.5 mg/ml in 50 mM Tris–HCl buffer (pH 8.0) containing 150 mM NaCl. The sample droplet was placed on a freshly cleaved mica or mica treated with 0.1% 3-aminopropyltriethoxysilane (APTES-mica). Bare mica surface was used as the substrate for imaging of the side views of the PbaA/PF0014, the PbaA/Trx-PF0014 and the PbaA_CΔ30_/PF0014 complexes, while the APTES-mica was used for top-view imaging of the PbaA pentamer and PF0014.

### DSC

DSC experiments were performed on a MicroCal VP-DSC instrument (Malvern Panalytical, Malvern, UK) using PbaA and the PbaA/PF0014 complex. Before scanning, the samples were degassed for 20 min at 5 °C. All sample solutions were scanned at a rate of 60 °C/hour in a temperature range of 10 °C–110 °C with a filtering period of 8 s, a pre-scan thermostat set for 15 min, and a post-scan thermostat set for 0 min. Twenty scans were obtained for each sample. Data were analyzed using the Origin software package.

### EM

The PbaA/PF0014 and PbaA/Trx-PF0014 complexes were dissolved at concentrations of 1.0 mg/ml in 50 mM Tris–HCl buffer (pH 8.0) containing 150 mM NaCl and subjected to negative stain EM analyses according to a previously described protocol^14,26^. In brief, the specimens stained with 2% uranyl acetate on the grid were observed using a 200-kV electron microscope (JEM-2200FS, JEOL Inc.) equipped with a DE20 direct detector CMOS camera (Direct Electron LP). Omega-type energy filtering was used for the data collection with a slit width of 15 eV. The particles images were collected at a nominal magnification of 30,000× (1.992 Å/pixel on the specimen). Total electron dose of 20 e−/Å^2^ was used for collecting 75 frames per image. The collected frames were merged after motion correction by a script provided by the manufacturer. The image processing was performed using Relion 3.0 software^27^ and reconstructed at 3D. The 3D renderings of all the maps were performed by UCSF Chimera^22^.

The PbaA_CΔ30_/PF0014 complex was used for structural analysis at a higher resolution using cryo-EM. A 2.5-μL aliquot was placed onto an R 1.2/1.3 Quantifoil grid (Quantifoil Micro Tools) pre-coated with a thin carbon film and pre-treated by a glow discharge. Plunge-freezing of the specimen was performed at 4 °C and 95% humidity using a Vitrobot Mark-IV (Thermo Fisher Scientific). The frozen grids were kept in cryo-storage in liquid nitrogen temperature until use. For data collection, the grid was loaded into a JEM-2100F electron microscope equipped with a 200-kV field emission electron source using a Gatan 626 cryo-specimen holder. A total of 350 images were acquired on a Gatan K2 Summit direct electron detector at a nominal magnification of 40,000×, corresponding to 0.93 Å per pixel on the specimen. The images were corrected for beam-induced motion with dose-weighting in Relion 3.0^27^, and their contrast transfer functions were estimated using CTFFIND4^28^. A subset of micrographs was used for particle picking with Gautomatch (https://www.mrc-lmb.cam.ac.uk/kzhang/Gautomatch/). The best classes from the 2D classification of this dataset were used for Relion internal autopicking across all micrographs, totaling 146,111 particles. After 2D classification, 45,807 particles were carried forward. The good classes, containing 15,117 particles, were used to generate five 3D models in Relion by imposing D5 symmetry. The best model was used as a reference for following 3D refinement. After contrast transfer function and data refinements, the final 3D cryo-EM map (EMD-0755) was reconstructed using 10,251 particles at a resolution of 7.3 Å with D5 symmetry and 8.5 Å with C1 symmetry using B-factors of −200 and −266, respectively [gold standard Fourier shell correlation (GS-FSC) criterion^29^].

For mapping the position of the N-terminus of PbaA, specimens of N-terminally hexahistidine-tagged PbaA protein in the complex with Trx-PF0014 were treated with an excess amount of a monoclonal antibody directed against the histidine tag (R&D Systems, Inc.) for 2 h at 4 °C. The sample of PbaA/Trx-PF0014 complex with the antibody was plunge-frozen on the EM grid and stored in liquid nitrogen storage as described above. The frozen grid was subjected to data collection and image processing using the same procedure as cryo-EM, except for the electron microscope (JEM-2200FS) and CMOS camera (DE20). The final 3D cryo-EM map was reconstructed at 14 Å resolution with 4,078 particles by imposing C1 symmetry.

### Solution scattering SANS/SAXS

SEC-SAXS measurements of the PbaA/PF0014 complex were obtained on Photon Factory BL-15A2 (Tsukuba, Japan) using UPLC ACQUITY (Waters) integrated with a SAXS set-up. The wavelength of the X-ray and the sample-to-camera distance were set to 1.21 Å and 2565.6 mm, respectively. In each measurement, 50 μL of 6 mg/ml proteins was loaded onto a Superdex 200 Increase 10/300 GL (GE Healthcare) pre-equilibrated with 50 mM Tris–HCl buffer (pH 8.0) containing 150 mM NaCl at a flow rate of 0.5 ml/min. During the elution of proteins, the flow rate was reduced to 0.10 ml/min. X-ray scattering was collected every 10 s on a PILATUS3 2 M detector over a range of scattering vector from *q*_min_ = 0.00625 Å^−1^ to *q*_max_ = 0.20 Å^−1^. The UV spectra at 280 nm were recoded every 10 s. The observed SAXS intensity was corrected for background, empty cell and buffer scatterings, and transmission factors and subsequently converted to the absolute scale using SAngler^30^.

For iCM-SANS experiments, a complex of 3 mg/ml of non-deuterated PbaA/75%-deuterated PF0014 as well as a complex of 75%-deuterated PbaA/75%-deuterated PF0014 was dissolved in 99.8% D_2_O (ISOTEC) containing 50 mM Tris–HCl (pH 8.0) and 150 mM NaCl to reduce the incoherent scattering and obtain high-quality statistical data in the higher *q*-range. SANS experiments were performed using Quokka installed at the Australian Centre for Neutron Scattering (ANSTO, Sydney, Australia). The SANS intensities were observed with 5.0-Å neutrons and at two sample-to-detector distances of 12.0 and 1.34 m, covering the *q*-range of 0.01–0.20 Å^−1^. The temperature was maintained at 25 °C during irradiation. The observed SANS intensity was corrected for background, empty cell and buffer scatterings, and transmission factors and subsequently converted to the absolute scale. For scattering measurements, the scattering intensity was normalized by the weight concentration of samples.

### Atomic structure modeling

The modeling of the PF0014 structure was performed by the global optimization method called conformational space annealing (CSA)^31,32^, which has been successfully used for protein structure modeling^33,34^. The segmented monomer map of PF0014 was matched to the PF0014 model. The PF0014 monomer map was segmented and extracted from the cryo-EM map of the PbaA/PF0014 complex using the Segger module^35^ implemented in UCSF Chimera^22^. The cross-correlation coefficient of the cryo-EM map and the model structure was used as a restraint term and augmented into the previously published energy function for protein structure modeling by CSA^33^. To reduce computational consumption, the initial models were fitted to the PF0014 monomer map by PowerFit^36^ and newly generated models were aligned with their parent models during CSA. A total of one hundred PF0014 monomeric models were finally obtained, and the best PF0014 monomeric model with the lowest energy function value was selected for building the complex model.

To construct the PbaA_CΔ30_/PF0014 complex model, the PbaA_CΔ30_ pentameric model was built from the crystal structure of PbaA (PDB ID: 3WZ2) by deleting the C-terminal residues at 212–231. The PF0014 decameric model was generated from the fitted PF0014 monomeric model by applying D5 symmetry. Finally, the complex model constituting ten PbaA_CΔ30_ and ten PF0014 protomers was constructed by fitting the two PbaA_CΔ30_ pentameric models and five PF0014 dimeric models into the cryo-EM map of the PbaA_CΔ30_/PF0014 complex, and the model was further refined through the Phenix real-space refinement procedure^37^. The structural quality of the model and goodness-of-fit to the cryo-EM map were measured by the Molprobity score^21^ and the cross-correlation coefficient of UCSF Chimera^22^, respectively.

## Supplementary information

Supplementary Information.

Supplementary Movie S1.

Supplementary Movie S2.

Supplementary Movie S3.

Supplementary Movie S4.

Supplementary Movie S5.

## Data Availability

The 3D cryo-EM density map of the PbaA_CΔ30_/PF0014 complex has been deposited in the Electron Microscopy Data Bank under accession number EMD-0755.
