# Supplementary material for: Supramolecular tholos-like architecture constituted by archaeal proteins without functional annotation
Source: Sci Rep. 2020 Jan 30;10:1540. doi: 10.1038/s41598-020-58371-2 (PMC6992696; doi:10.1038/s41598-020-58371-2)
Supplement: Supplementary file 1 — Supplementary Information. [file 41598_2020_58371_MOESM1_ESM.pdf]

Supplementary Information

Supramolecular tholos-like architecture constituted by archaeal proteins without functional annotation

Maho Yagi-Utsumi[1,2,3,4], Arunima Sikdar[2,3], Chihong Song[5], Jimin Park[6], Rintaro Inoue[7], Hiroki Watanabe[1], Raymond N. Burton-Smith[5], Toshiya Kozai[8], Tatsuya Suzuki[1], Atsuji Kodama[1], Kentaro Ishii[1], Hirokazu Yagi[4], Tadashi Satoh[4], Susumu Uchiyama[1,9], Takayuki Uchihashi[1,8], Keehyoung Joo[10], Jooyoung Lee[6, 10, 11], Masaaki Sugiyama[7], Kazuyoshi Murata[3,5], and Koichi Kato[1,2,3,4,*]

[1]Exploratory Research Center on Life and Living Systems (ExCELLS), National Institutes of Natural Sciences, Okazaki, Aichi 444-8787, Japan

[2]Institute for Molecular Science (IMS), National Institutes of Natural Sciences, Okazaki, Aichi 444-8787, Japan

[3]SOKENDAI (The Graduate University for Advanced Studies), Okazaki, Aichi 444-8787, Japan

[4]Graduate School of Pharmaceutical Sciences, Nagoya City University, Nagoya, Aichi 467-8603, Japan

[5]National Institute for Physiological Sciences, National Institutes of Natural Sciences, Okazaki, Aichi 444-8787, Japan

[6] School of Computational Sciences, Korea Institute for Advanced Study, Seoul 02455, Republic of Korea

[7]Institute for Integrated Radiation and Nuclear Science, Kyoto University, Kumatori, Osaka 590-0494, Japan

[8] Department of Physics, Nagoya University, Nagoya, Aichi 464-8602, Japan

[9] Department of Biotechnology, Graduate School of Engineering, Osaka University, Suita, Osaka 565-0871, Japan

[10]Center for Advanced Computation, Korea Institute for Advanced Study, Seoul 02455, Republic of Korea

[11]Beijing Computational Science Research Center, Haidian District, Beijing, 10084, China

* Author to whom correspondence should be addressed.

Supplementary Figures

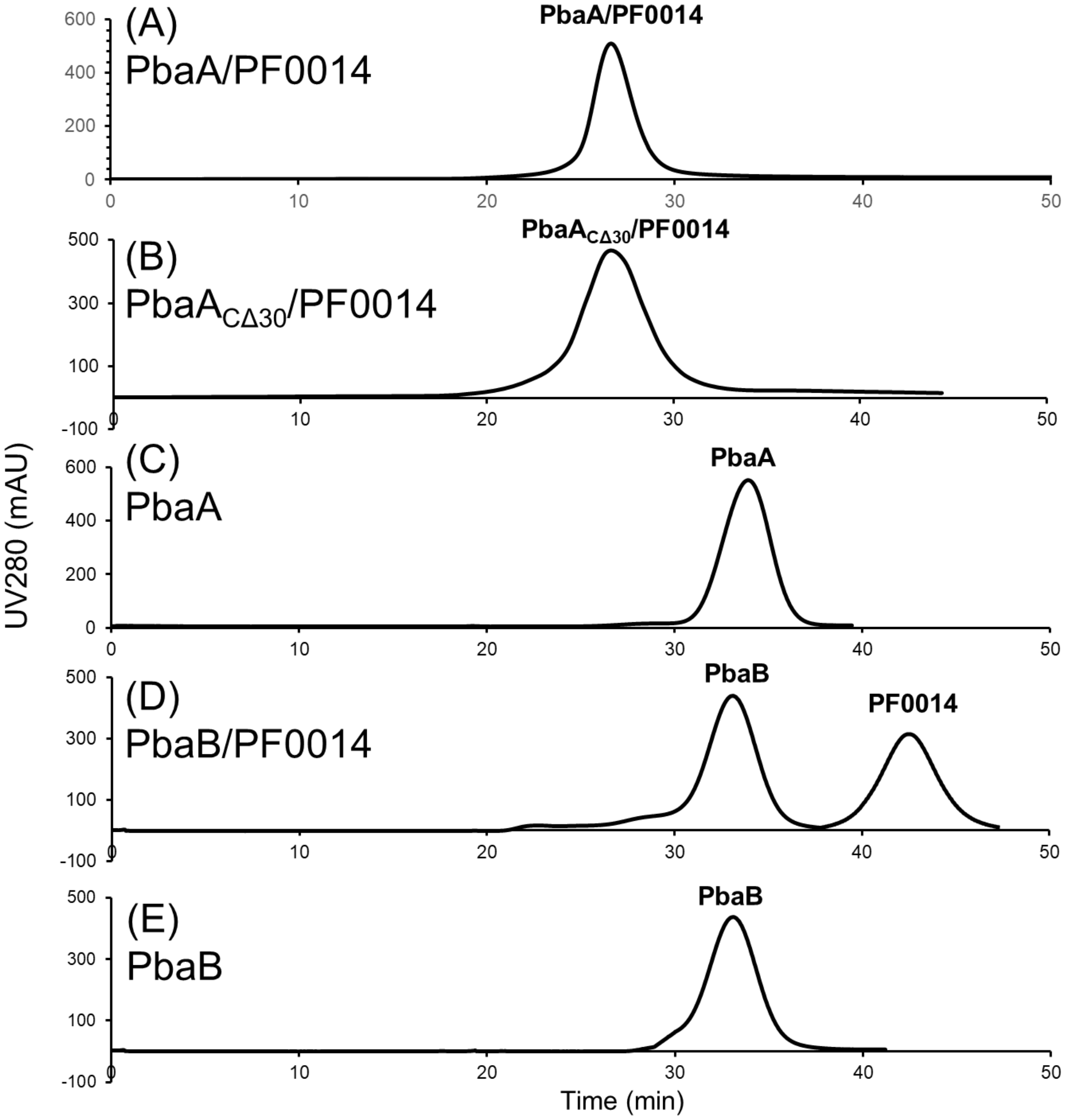

Supplementary Figure S1: SEC profiles of mixtures of (A) PbaA and PF0014 at a 1:1 molar ratio, (B) PbaA $_{C\Delta30}$ and PF0014 at a 1:1 molar ratio, (C) PbaA, (D) mixtures of PbaB and PF0014 at a 1:1 molar ratio, and (E) PbaB. SEC was performed using a HiLoad Superdex 200 column (GE Healthcare) at a flow rate of 2.0 ml/min.

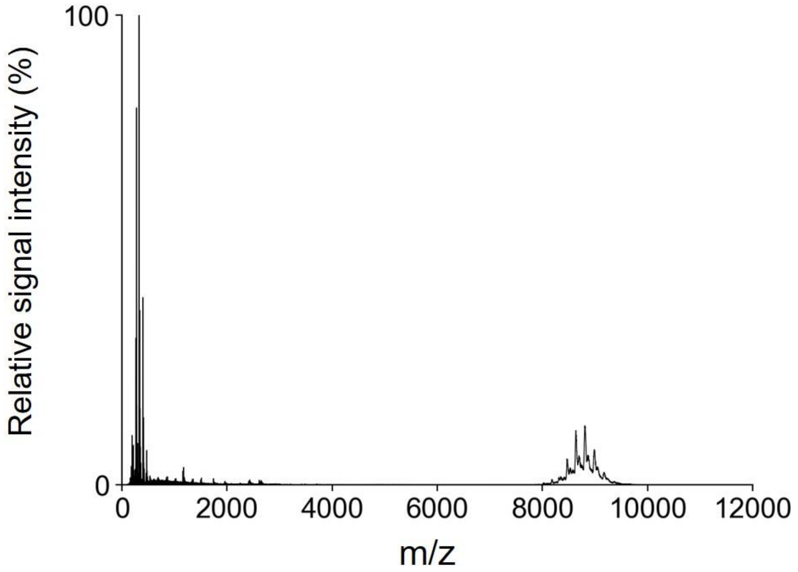

Supplementary Figure S2: A whole m/z range mass spectrum of the mixtures of PbaA and PF0014 at a 1:1 molar ratio under a non-denaturing condition.

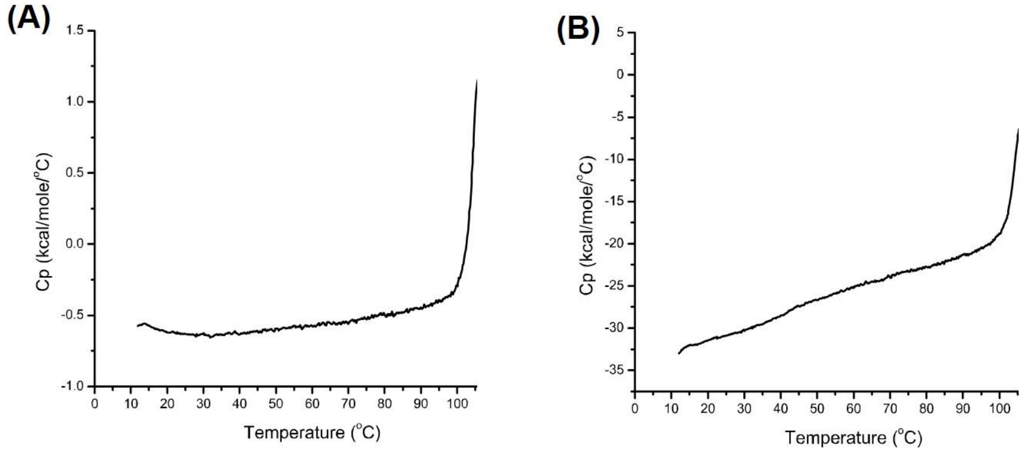

Supplementary Figure S3: DSC thermograms of (A) PbaA and (B) the PbaA/PF0014 complex.

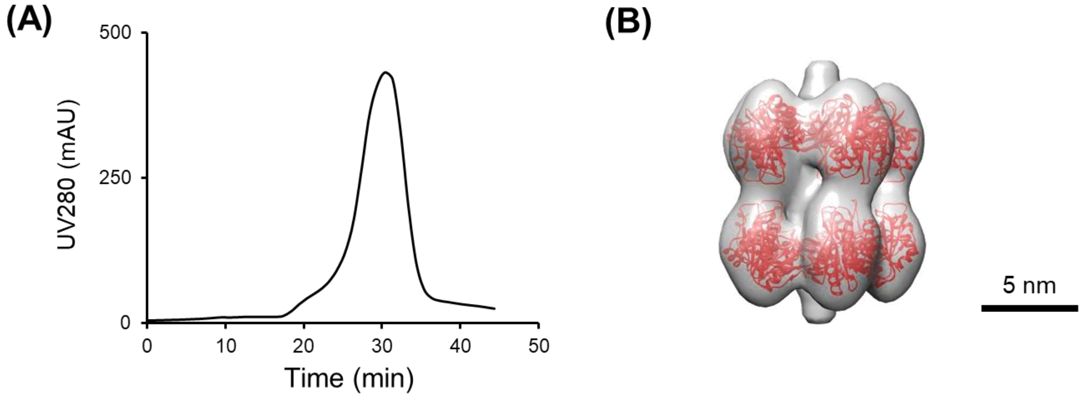

Supplementary Figure S4: (A) SEC profiles of $PbaA_{C\Delta30}$. SEC was performed using a HiLoad Superdex 200 column (GE Healthcare) at a flow rate of 2.0 mg/ml. (B) The double-pentameric ring structure of $PbaA_{C\Delta30}$ obtained by single particle negative stain EM. The crystal structure of the $PbaA_{C\Delta30}$ was superimposed onto the EM map.

Supplementary Table S1: Negative stain-EM and cryo-EM data collection and processing

	**PbaA/PF0014** (Figure 3)	**PbaA/Trx-PF0014** (Figure 5A-B)	**the hexahistidine-tagged PbaA/Trx-PF0014 complex with a monoclonal antibody** (Figure 5C-D)	**$PbaA_{C\Delta30}$/PF0014** (Figure 6)
Method	Negative stain-EM	Negative stain-EM	Cryo-EM	Cryo-EM
Microscope	JEM-2200FS	JEM-2200FS	JEM-2200FS	JEM-2100F
Detector	DE-20	DE-20	DE-20	K2 Summit
Sampling frequency	1.992 Å/pixel	1.992 Å/pixel	1.09 Å/pixel	0.93 Å/pixel
Micrographs acquired	61	59	119	350
Particles contributing to final map	1,206	1,384	4,078	10,251
Applied symmetry	D5	D5	C1	D5/C1
Defocus range	2-4 μm undefocus	2-4 μm underfocus	1-3 μm underfocus	1-3 μm underfocus
Software used	RELION2	RELION2	RELION2	RELION 3
Resolution	17 Å	17 Å	14 Å	7.3 Å (D5 symmetry)<br>8.5 Å (C1 symmetry)

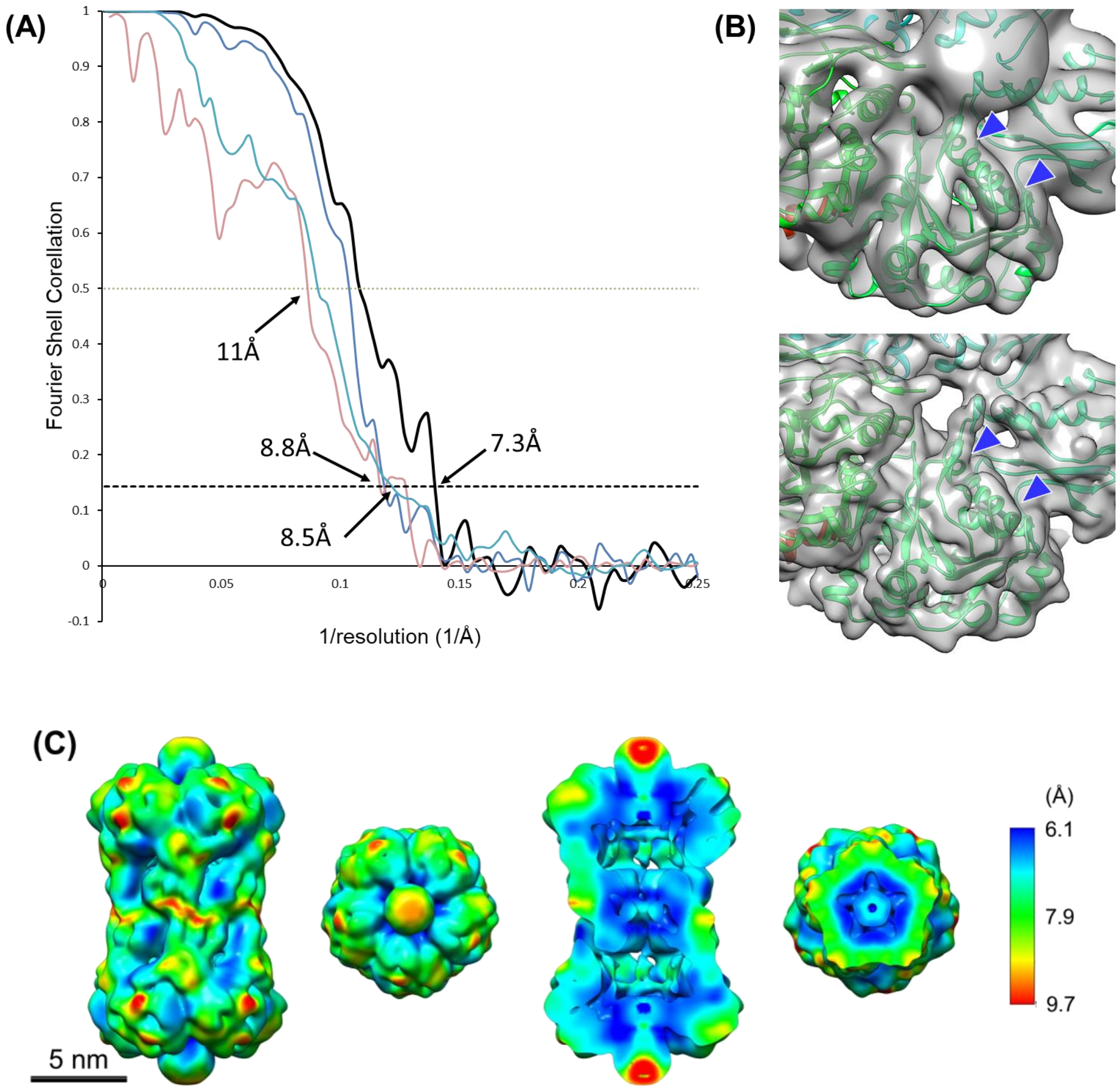

Supplementary Figure S5: (A) Gold standard Fourier shell correlation curves of the PbaA$_{C\Delta30}$/PF0014 complex reconstruction. With D5 symmetry, the final sharpened soft-masked map (black line) corresponds to 7.3 Å at FSC=0.143, the correlation of the two unmasked maps (blue line) corresponds to 8.8 Å at FSC=0.143, and the correlation between map and model (red line) corresponds to 11 Å at FSC=0.5. With C1 symmetry (no symmetry imposed), the global estimated resolution is 8.5 Å at FSC=0.143 (cyan line). (B) Close-up views of the PbaA$_{C\Delta30}$/PF0014 complex reconstruction displayed as surface representation countered at 5.5σ (upper) and a map simulated from the model structure of the PbaA$_{C\Delta30}$/PF0014 complex (lower). The surface models were superimposed on the simulated structure model of the PbaA$_{C\Delta30}$/PF0014 complex. This demonstrates evident densities corresponding to α-helices (as exemplified by that

marked with blue arrowheads). Simulated map from the model structure showed similar structural features. (C) The surface representations (side and top views) (left panels) and the centrally-sliced images (side and top views) (right panels) of the PbaA$_{C\Delta30}$/PF0014 complex contoured at 3σ are illustrated with local resolution calculated by the bsoft bLocRes module [Heymann, J.B. & Belnap, D.M. Bsoft: Image processing and molecular modeling for electron microscopy. *J. Struct. Biol.* **157**, 3-18, doi:10.1016/j.jsb.2006.06.006 (2007)].

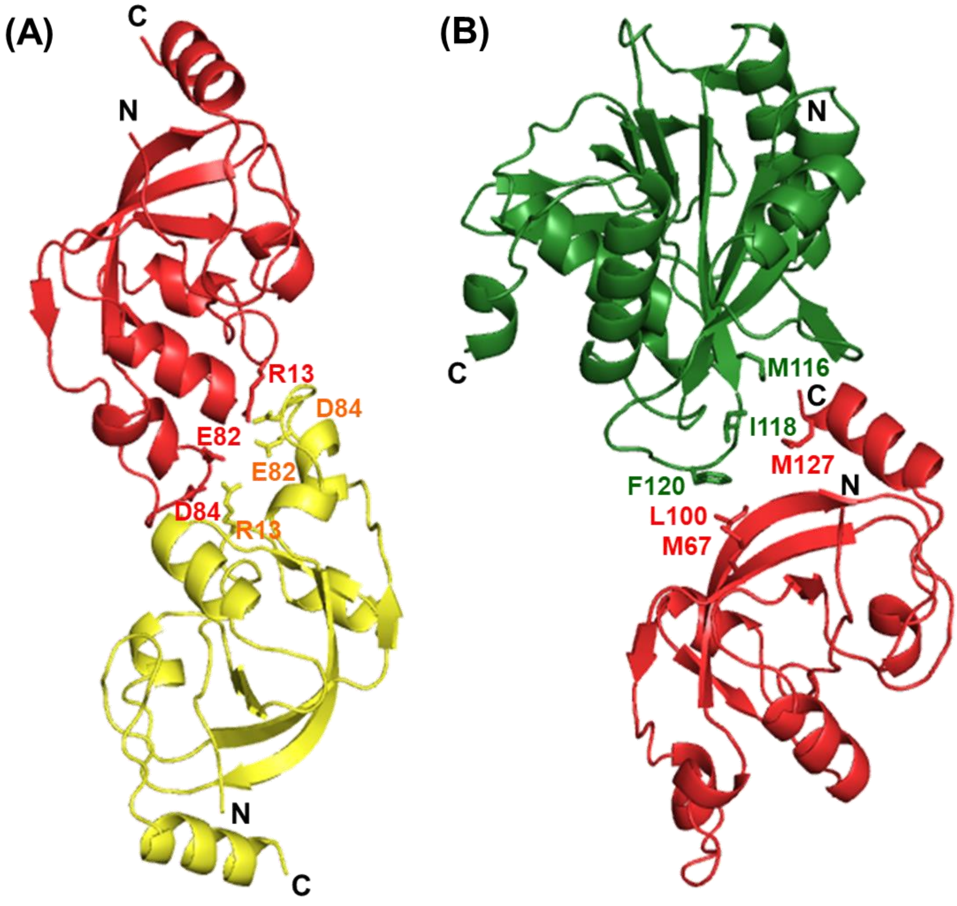

Supplementary Figure S6: Ribbon models of (A) a PF0014 homodimer and (B) a PbaA$_{C\Delta30}$ protomer (green) interacting with a PF0014 protomer (red). The amino acid resides located on the interface are labeled and shown in stick models.

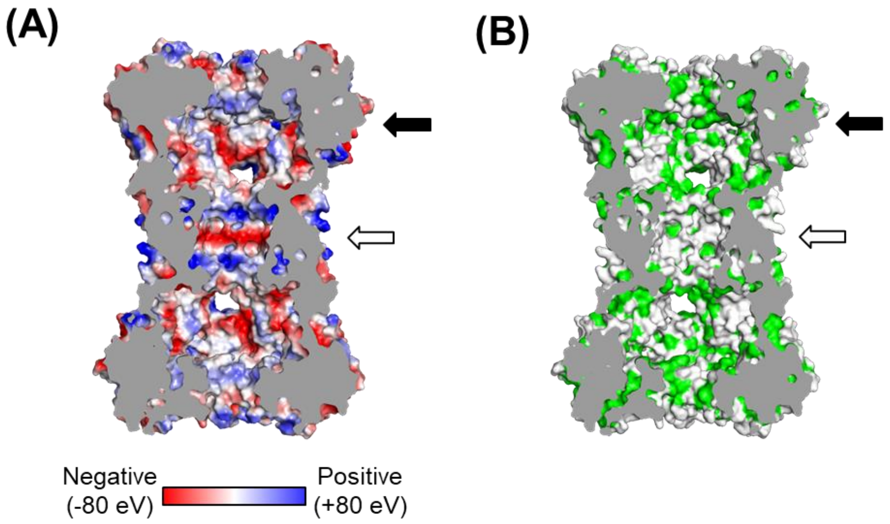

Supplementary Figure S7: Long axis cross-section of the model of the PbaA$_{C\Delta30}$/PF0014 complex shown with (A) electrostatic potential and (B) hydrophobic surfaces (green). Black and white arrows indicate the interfaces between PbaA$_{C\Delta30}$ and PF0014 and between two PF0014 protomers, respectively. Electrostatic potential was calculated and visualized using the UCSF Chimera software.

Supplementary Movie S1: The PbaA/PF0014 complex

Supplementary Movie S2: PbaA

Supplementary Movie S3: PF0014

Supplementary Movie S4: The PbaA/Trx-PF0014 complex

Supplementary Movie S5: The PbaA$_{C\Delta30}$/PF0014 complex
